# Identification and isolation of Genotype-I Japanese Encephalitis virus from encephalitis patients

**DOI:** 10.1186/1743-422X-7-345

**Published:** 2010-11-26

**Authors:** Lihua Wang, Shihong Fu, Hailin Zhang, Xufang Ye, Deshan Yu, Zhang Deng, Jun Yuan, Yougang Zhai, Minghua Li, Zhi Lv, Weixin Chen, Hongyue Jiang, Xiaoyan Gao, Yuxi Cao, Huanyu Wang, Qing Tang, Guodong Liang

**Affiliations:** 1 State Key Laboratory for Infectious Disease Prevention and Control (SKLID), Institute for Viral Disease Control and Prevention, China CDC, Beijing 100052, PR China; 2 Yunnan Institute of Endemic Diseases Control and Prevention, Dali City, 67100, PR China; 3 Guizhou Center for Disease Control and Prevention, Guiyang 550001, PR China; 4 Gansu Center for Disease Control and Prevention, Lanzhou, 730000, PR China

## Abstract

Historically, Japanese Encephalitis virus (JEV) genotype III (GIII) has been responsible for human diseases. In recent years, JEV genotype I (GI) has been isolated from mosquitoes collected in numerous countries, but has not been isolated from patients with encephalitis. In this study, we report recovery of JEV GI live virus and identification of JEV GI RNA from cerebrospinal fluid (CSF) of encephalitis patients in JE endemic areas of China. Whole-genome sequencing and molecular phylogenetic analysis of the JEV isolate from the CSF samples was performed. The isolate in this study is highly similar to other JEV GI strains which isolated from mosquitoes at both the nucleotide and deduced amino acid levels. Phylogenetic analysis based on the genomic sequence showed that the isolate belongs to JEV GI, which is consistent with the phylogenetic analysis based on the pre-membrane (PrM) and Glycoprotein genes. As a conclusion, this is the first time to isolate JEV GI strain from CSF samples of encephalitis patients, so continuous survey and evaluate the infectivity and pathogenecity of JEV GI strains are necessary, especially for the JEV GI strains from encephalitis patients. With respect to the latter, because all current JEV vaccines (live and inactivated are derived from JEV GIII strains, future studies should be aimed at investigating and monitoring cross-protection of the human JEV GI isolates against widely used JEV vaccines.

## Findings

Japanese encephalitis (JE) is one of the leading causes of acute encephalopathy affecting children and adolescents [1]. With the spread of JEV into new areas and the potential for further expansion, JE has become a worldwide public health problem [1-3]. Almost half of the human population currently lives in countries where JEV occurs and nearly 50,000 cases of JE occur worldwide and 15,000 of them die per year [1-3]. JEV, as the etiologic agent of JE, has been subdivided into five genotypes [4]. The majority of JEV isolates from humans were genotype III as reported previously [2-4]. Over the last two decades JEV GI strains has been isolated from mosquitoes or swine collected in Southeast Asia, Australia, Korea, Japan and China, etc. [4-9]. There are few JEV GI isolates from JE patients, so there is no evidence showing that JEV GI associated with human encephalitis.

**Figure 1:**
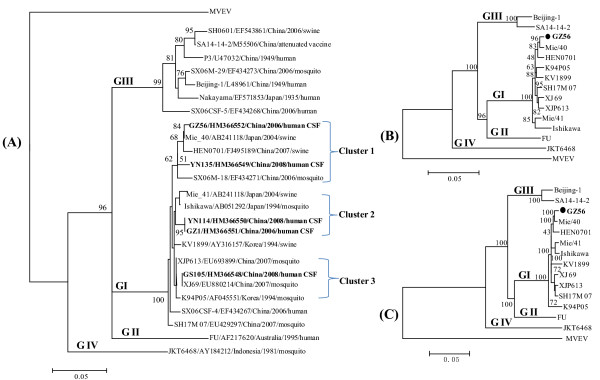
**Phylogenetic analysis of JEV strains in CSF from JE patients, China**. (A) Phylogenetic analyses based on the PrM gene of JEV; (B) Phylogenetic analyses based on the E gene of JEV; (C) Phylogenetic analyses based on the full-length genome of JEV. Phylogenetic analyses were performed by the neighbor-joining method using MEGA version 4 http://www.megasoftware.net. Bootstrap probabilities of each node were calculated with 1,000 replicates. The tree was rooted by using Murray Valley encephalitis virus (MVEV) sequence as the out group virus. Horizontal branch lengths are proportional to genetic distance and vertical branch lengths have no significance. The scale indicates the number of nucleotide substitutions per site. Sequences from this study are in boldface. Viruses were identified by using the nomenclature of virus strain/GenBank number/country/year of isolation/origin as showed in Figure 1(A).

In this study, the acute (1-3 days after onset) serum and CSF specimens of patients with a clinical diagnosis of JE but negative for JEV-specific IgM antibody testing were obtained for diagnosis purposes from JE surveillance laboratories in Yunnan (250 cases), Guizhou (120 cases) and Gansu (50 cases) provinces of China in 2006 and 2008. These specimens were stored at -80°C and transported on dry ice to Institute for Viral Disease Control and Prevention (IVDCP), China CDC, for JEV serological examination, JEV nucleic acid detection and virus isolation. First, the samples were re-screened to verify the absence of JEV-specific IgM antibody using the JEV IgM-Capture ELISA kit (Shanghai B & C Enterprise Development Co. Ltd, Shanghai, PR China) [10]. Of those which did not contain detectable JEV IgM antibody were performed for viral RNA extraction directly by using the QIAamp viral RNA extraction kit (QIAGEN, Valencia, CA, USA) without risk of altering the genome by passage in vitro. cDNA was synthesized using random hexmer primer with Ready-To-Go You-Prime First-Strand Beads (Amersham Pharmacia Biotech, Piscatawy, NJ, USA), and a hemi-nested PCR procedure was used to amplify a 492-bp fragment of the premembrane (PrM) gene of JEV by using the Takara LA Taq PCR kit (Takara Bio Inc., Shiga, Japan). The primers which covered all the JEV genotypes were derived from Ishikawa strain genome sequences [GenBank:AB051292]; PrMF: 5'-CGT TCT TCA AGT TTA CAG CAT TAG C-3' (251nt-275nt), PrMR1: 5'-CG Y TTG GAA TGY CTR GTC CG-3' (724nt-743nt), and PrMR2: 5'-CCY RTG TTY CT G CCA AGC ATC CAM CC-3' (901nt-925nt). PCR products were purified with the QIAquick PCR Purification Kit (Qiagen), and then the purified products were sequenced with the BigDye1 Terminator v1.1 Cycle Sequencing Kit (Applied Biosystems) on ABI 3130 Genetic Analyzer (Applied Biosystems). Multiple sequence alignments and phylogenetic analysis were done using ClustalX version 2.0 http://www.clustal.org and MEGA version 4 http://www.megasoftware.net by the neighbor-joining method with bootstrap probabilities of 1,000 replicates. Totally five CSF samples (Table 1) were tested positive by RT-PCR, and none for the serum samples. The sequences [GenBank: HM366548-HM366552] amplified from CSF samples are clustered within GI (Figure 1) by phylogenetic analysis based on the 240 nucleotide (nt) sequence of the JEV prM gene. Aliquots of CSF samples which showed positive by RT-PCR were inoculated onto BHK-21 cells for virus isolation. One isolate (GZ56) was obtained, which was taken from a 0.5 years old female patient residing in Guizhou province (Table 1). The complete genomic sequence [GenBank:HM366552] of GZ56 strain was then determined by RT-PCR and sequencing using flavivirus-specific primers [11] and overlapping primers designed from the sequence of JEV strain Ishikawa. The genome of GZ56 strain has identical size of 10,965 nt with a 96-nt 5' nontranslated region (NTR) and a 570-nt 3' NTR, and has the same genomic structure with other JEV strains. The single open reading frame (10, 296 nt) codes for a polyprotein of 3, 432 amino acid (aa). Phylogenetic analysis based on the genomic sequence showed that GZ56 strain belongs to GI (Figure 1), which is consistent with the phylogenetic analysis based on the PrM and E genes (Figure 1). GZ56 strain showed high homology with JEV GI strains obtained from swine of Japan (Mie/40) and China (HEN0701) in nt (99.2% and 98.2% respectively) and aa (99.8% and 99.5% respectively).

**Table 1:** Results of IgM, RT-PCR and virus isolation to detect evidence of JEV in 1 clinical samples from encephalitis patients.

Patient	Age(y)	Sex	Onset time	Clinical diagnosis	Interval between onset and sampling(d)	Sample type	Laboratory diagnosis		Genotype
								
							IgM	RT-PCR	
GZ56*	0.5	F	11/08/2006	JE	1 1	S C	- -	- +	GI
GZ1	13	M	24/08/2006	JE	1 1	S C	- -	- +	GI
GS105	12	F	10/05/2006	JE	2 2	S C	- -	- +	GI
YN114	28	M	01/07/2008	JE	3 3	S C	- -	- +	GI
YN135	37	M	12/07/2008	JE	3 3	S C	- -	- +	GI

Abbreviations: C, cerebrospinal fluid; S, serum; F, female; M, male; GZ, Guizhou province; GS, Gansu province; YN, Yunnan province; "+", positive; "-", negative.

* Virus strain GZ56 was obtained from patient GZ56.

The five patients who showed JEV GI positive by RT-PCR or virus isolation include both male and female with age range from 0.5 to 37 years old (Table 1), and resided in southwestern (Yunnan and Guizhou province) and northwest areas (Gansu province) of China, within latitude 24°37'~ 42°57'N and longitude 92°13 '~ 111°15'E. These areas are known to be endemic for JE, and JEVs have been isolated from mosquitoes collected in these areas [12,13]. Molecular epidemiological study showed that all of the JEV isolates obtained either from mosquitoes or from clinical samples of human beings before 1970 s were GIII in China [6,9,12,13]. JEV GI isolates was first obtained from mosquitoes collected in China in 1977, thereafter JEV GI isolates from mosquitoes showed gradually increasing frequency in China including Yunnan, Guizhou and Gansu provinces, which suggests that JEV GI is replacing JEV GIII and is becoming the major genotype in these areas in recent years [6,9,12,13]. In our study, JEV GI strain was isolated for the first time from CSF samples of JE patients in China. Previously, partial sequences of JEV GI were detected in specimens of meningitis patients in Japan [14] and in JE patients in an outbreak of China in 2006 [10]. These results showed that JEV GI associated with human encephalitis. JEV GI isolates from the patients in this study are classified into 3 clusters (Figure 1), and closely related to the recently identified JEV GI strains including JEV GI strains identified during JE outbreak of Shanxi province of China in 2006 [10]. One from northwest area was grouped within cluster 3, the others from southwestern areas were grouped in both cluster 1 and 2. The mechanism for JEV GI spread into southwestern and northwest areas of China needs further investigation.

In this study, all samples (CSF and serum) from five cases were negative for JEV IgM antibody examined by laboratories of local hospital, but based on clinical features, living area (in JE endemic region) of the patients, and cases happened in the season of JE, the five cases were still diagnosed as JE by the local clinicians. Further investigation in our laboratory showed that CSF samples of the patients were positive for JEV GI (Table 1), which confirmed that the diagnosis of local clinicians is correct. The data indicated that the diagnosis of GI JEV infection using acute samples should perform RT-PCR detection, especially for the acute samples which showed absence of JEV IgM antibody to reduce misdiagnosis in JE endemic areas. As a conclusion, this study is the first time to obtain JEV GI isolates from encephalitis patients in China, continuous survey and evaluate the infectivity and pathogenecity of JEV GI strains, especially for the JEV GI strains from humans, are necessary. In addition, because all current JEV vaccines (live and inactivated are derived from JEV GIII strains [1-3], the investigation and monitor of cross-protection between the JEV GI strains and widely used JEV GIII vaccines are needed.

## Competing interests

The authors declare that they have no competing interests.

## Authors' contributions

LHW and SHF carried out serological examination, nucleic acid detection and sequencing, participated in the sequence alignment, phylogenetic analysis and drafted the manuscript. HLZ, XFY and DSY participated in the collection of clinical samples. ZD, JY, YGZ, MHL, ZL, WXC, HYJ, XYG, YXC, HYW and QT participated in the serological studies, virus isolation, and the design of the study. GDL conceived of the study, and participated in its design and coordination. All authors read and approved the final manuscript.

## Authors' information

Dr. Lihua Wang, Ph.D., is an associate professor at the State Key Laboratory for Infectious Disease Prevention and Control, the Institute for Viral Disease Control and Prevention, Chinese Center for Disease Control and Prevention. His current research focuses on medical microbiology, detection and diagnosis of emerging infectious pathogens.
